# Microalgae toxins in food products and impact on human health: a review

**DOI:** 10.3389/fnut.2025.1603843

**Published:** 2025-07-11

**Authors:** W. A. T. N. Withana, D. M. D. I. Gunarathna, D. M. G. I. Dissanayake, A. I. Rathnayake, H. K. S. de Zoysa, Viduranga Y. Waisundara

**Affiliations:** ^1^Department of Bioprocess Technology, Faculty of Technology, Rajarata University of Sri Lanka, Anuradhapura, Sri Lanka; ^2^Australian College of Business and Technology – Kandy Campus, Kandy, Sri Lanka

**Keywords:** microalgae, toxins, food products, impact, human health

## Abstract

Microalgal toxins are secondary metabolites synthesized by cyanobacteria, *dinoflagellates*, and diatoms in response to environmental stress. Humans and animals can be exposed to these toxic compounds through food, water, and aerosolized toxins and these toxic compounds are capable of causing acute and chronic health issues like paralysis, liver damage, cancer, and even death by employing several molecular mechanisms such as sodium channel blocking, protein phosphatase inhibition, cellular membrane disruption etc. Microalgal toxin poisoning through food products is a major concern as microalgae are largely consumed as dietary supplements. These toxins can easily bioaccumulate and be biomagnified via food chains. Hence, proper screening and quality control measures for these microalgal toxins should be implemented. Cytotoxins, dermatoxins, neurotoxins, hepatotoxins, and endotoxins are the main toxins produced by the microalgae. Microalgae are effectively incorporated into the food industry in a diverse range. Toxic contaminants from the microalgae are a silent threat to food security and human health. There are some regulatory models when consuming microalgae-related food products and water due to their toxic effects. Detecting the toxins in the initial stage, studying the impact of toxin production due to environmental factors, and developing effective mitigation strategies to ensure food safety, is a future needs in this field.

## 1 Introduction

Microalgae are a diverse group of unicellular, photosynthetic microorganisms (1) that act as the primary producers in aquatic ecosystems (2). Their nutrient richness and the ability to synthesize bioactive compounds are considered highly beneficial as they can be used in various industries, such as food and nutraceuticals (3), medicines (4), cosmetics (5), animal feed (6), agriculture (7), and biofuel production (8). By 2050, it is estimated that the global population will reach 9.7 billion, requiring a doubling of global food production to satisfy the rising food demand (9). Microalgae have been consumed as a food source for thousands of years (9) and are loaded with essential nutrients (10). Apart from that, their antibiotic (11), antioxidant (12), anti-viral (13, 14), anticancer (15, 16), anti-inflammatory (17) and neuroprotective properties (18) offer many health benefits by reducing and preventing the risk of developing diseases like cancer, macular degeneration, cataracts, type 2 diabetes, and cardiovascular diseases (19). Compared to conventional crop cultivation, microalgae cultivation offers numerous advantages, including continuous year-round output, less land consumption, better yields, etc. (1). Hence, as an alternative food source, microalgae is a promising solution. Currently, microalgae species including *Arthrospira platensis*, *Chlorella* spp., *Dunaliella salina*, *Aphanizomenon flos-aquae*, *Odontella aurita*, *Tetraselmis chuii*, *Haematococcus pluvialis*, *Schizochytrium* spp., and *Ulkenia* spp., are commercially cultivated for human consumption and issued to the market as tablets, pellets, powders, capsules, or in liquid form (1). Furthermore, microalgae-incorporated food items, such as cookies, sausages, cheese, and ice cream etc., are also available in the market (1).

Certain microalgal species can produce toxic compounds known as microalgal toxins, and some environmental factors like temperature, light intensity, and nutrient availability are believed to trigger the formation of harmful algal blooms (HABs) (20). As the population of toxin-producing microalgae increases within these blooms, they release larger quantities of toxic compounds such as Saxitoxin, Ciguatoxins, Nodularin, Anatoxin-a, and many more (21) into water bodies, leading to the complete disruption of the entire ecosystem. Moreover, these toxins can bioaccumulate through aquatic food webs in higher trophic levels, including humans, which eventually leads to detrimental chronic renal, cardiovascular, gastrointestinal, respiratory, and neurological disorders (22–24). Therefore, it is important to thoroughly examine microalgal toxin production and releasing mechanisms to develop monitoring and mitigation strategies to prevent food contamination.

When it comes to public health, there are three major ways of exposure to algal toxins: (1) consumption of toxin-contaminated food (25), (2) Inhalation of aerosolized toxins (26), and (3) Skin contact with toxin-containing liquids (27). Accordingly, the simplest way to experience microalgal toxin-associated poisoning is by consuming toxin-contaminated food and water (28). In the case of shellfish, as they are filter feeders, toxins such as saxitoxins or domoic acid can accumulate (29). Moreover, consuming dietary supplements such as *Spirulina* or *Chlorella*-based on some microalgae supplement, poses a risk of contaminated microalgal toxins (30). This can be due to contamination by toxin-producing species, even under commercial setups (30). Proper screening for toxic compounds and quality control measures should be implemented, as children, the elderly, pregnant women, and immunocompromised individuals consume these dietary supplements.

To minimize the contamination of food by microalgal toxins and to prevent their short- and long-term health implications, it is essential to have a clear understanding of the specific species responsible for producing these toxins, the factors that influence their production and release, their mode of action, and their occurrence in the human diet. In this review, we try to provide an overview of the types of microalgal toxins, how they enter into food chains, and their associated health implications, while understanding the molecular mechanisms underlying the production of these toxins and their role in developing life-threatening diseases. The consumer protections and regulatory models regarding the microalgae and their future directions.

## 2 Microalgae and toxins

### 2.1 Definition, classes, and biological characteristics of microalgae

**FIGURE 1:**
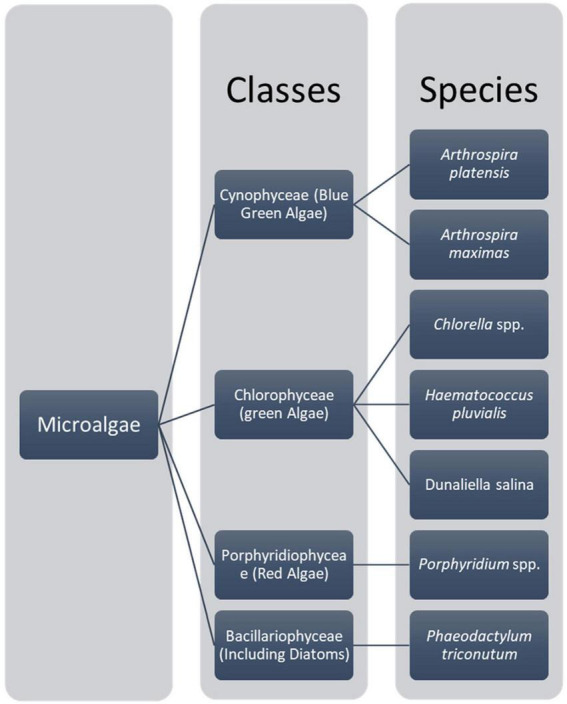
Main micro algae classes and their main species (32).

Microalgae are prokaryotic and primary photosynthetic eukaryotic, single-celled organisms that are phylogenetically and taxonomically divergent (31, 32). Algae can be classified as unicellular and multicellular according to their sizes and shapes (33, 34). These microalgae are in diverse habitats and can be found in almost all areas on earth, including different water bodies with fresh water, hypersaline environments, and sea water, rocks, or moist soil (31). The classification of microalgae can be based on various aspects such as morphological features, pigmentations, and photosynthetic membranes (32). As Torres et al. (31) describe, the most typical classification of microalgae is with classes Chlorophyceae [green algae, Cyanophyceae (blue-green algae), Chrysophyceae (golden algae), and Bacillariophyceae (Diatom)] (31). A chart summarizing the main microalgae classes with their main relevant species is shown in Figure 1. Consequently, microalgae are fast growers and highly productive even in a limited land area (35), doing photosynthesis and completing their whole lifecycle within a few days. Mostly, it needs simple nutrients and abundant sunlight for its survival (35). Mainly, microalgae are smaller in size; their sizes range from l μm to 1 mm and belong to a heterogenous group. *Chlorella*, which lives primarily in freshwater or soil, is 2 μm to 10 μm in diameter and spherical (36). Usually, microalgae are orthotropic, while some are mixotrophs. Their mechanism is different from the terrestrial plants as they do not have the same cell differentiation (36, 37). In 1830, color was first used to differentiate microalgae into green, brown, and red (38). However, recent studies mainly focused on phylogeny and molecular studies to analyze the structure and understand the relationship between algae and other organisms (39). Algae do not have a common ancestor, and they are called a polyphyletic group without a taxonomic value (31, 34, 40). According to the color pigments produced by the chloroplast, the color of microalgae comes from phycobiliproteins and chlorophylls (38). The Phylum cyanobacteria belongs to the prokaryotic cell microalgae, and eukaryotic species mainly consist of red microalgae (Rhodophyta), green microalgae (Chlorophyta), and diatoms (Bacillariophyta) groups (41, 42).

Microalgae are considered old living beings on the planet, and they exist in all of Earth’s ecosystems. They can live in adverse conditions like radiation, temperature, oxygen, pH, and salinity. Therefore, it can lead to a vast area of scientific research and exploration (34, 41, 43). Rationalizing microalgae by bioprospecting the new species, studying unique lineages of these organisms, and properly choosing microalgae and cultivating them is very important (44).

### 2.2 Toxins produced by microalgae and it’s mechanism action

**FIGURE 2:**
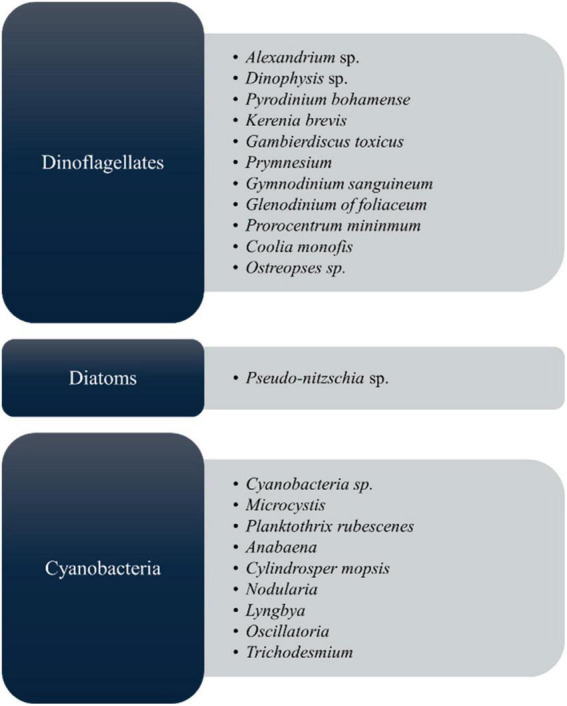
Mainly algal species that cause for harmful algal blooms (HABs) (39, 144).

As shown in Figure 2, microalgal species, including those belonging to the groups of cyanobacteria, diatoms, and *dinoflagellates*, produce toxic compounds known as microalgal toxins, which have harmful effects on both aquatic ecosystems and human health by mainly harmful algal blooms (HABs) (45). Each species generates a distinct array of algal toxins, characterized by varying chemical structures and toxic effects (25, 46). These toxins are produced as a defensive strategy to protect against predators in response to grazing pressure; hence, they play a major role in their life survival (47, 48). In the market, most biomedical exploration utilizes *Spirulina* and *Chlorella*, but it must ensure safety for the final commercial outcome. Manali et al. (49) showed that microcystin contamination was only detected in fish food supplements, not in the *Spirulina* ingredients dietary supplements (49). However, to this today, there are no considerable reports that *Arthrospira* spp. and *Chlorella* spp. produce toxins, therefore establishing their safety by the US Food and Drug Administration (US FDA) (50) by GRN No.127 (51–53). From the microalgal toxins, cyanotoxins are the most diverse group of natural toxins from the chemical and toxicological view (54). Because of the algae, it can cause the death of people, fish, and other living things. Most toxic microalgae are *dinoflagellates* and diatoms in marine and freshwater, and Figure 2 depicts the major toxic algal species. According to many records, microalgal toxins associated with food poisoning significantly impact public health (22). Most of the incidents are related to contaminated seafood consumption (55, 56) leading to acute food poisoning with distinct symptoms such as vomiting, diarrhea, numbness, confusion, memory loss, and in extreme cases, paralysis, brain and liver damage, and even death (25). Apart from that, long-term exposure to these toxins can cause damage to the gut, liver, and lung health (22).

Certain microalgae produce bioactive compounds known as phycotoxins, which are toxic substances generated by specific genera of *dinoflagellates*, diatoms, and cyanobacteria (57). The situation worsens as these toxins accumulate in water bodies, posing risks to humans, marine life, and aquatic organisms (58). This review examines how several well-known microalgae toxins function at the biochemical, molecular, and toxicological levels and analyzes their impact on the risk posed to the human population and their mechanism of action.

Cyanobacteria produce diverse toxins as secondary metabolites, which are hazardous to many other organisms. Researchers have discovered that these pollutants cause a significant threat to human health in diverse parts of the world. The main two types of toxins produced by cyanobacteria are cytotoxins and biotoxins (59, 60). Table 2 summarizes the cyanobacterial toxins produced by various types of blue-green algae and their impacts, as listed in the below.

#### 2.2.1 Cytotoxins

Cylindrospermopsin is a known cytotoxin. It is one of the toxins made by *Cylindrospermopsis mceberskii*, and it is the only alkaloid compound among the hepatotoxicants. The toxic effect of this compound is not only for the liver; it has been found to cause tissue destruction to the kidneys (61). Some marine cyanobacteria species produce this toxin, and it is closely related to the cholera toxin, but it is not so toxic to animals; however, it is lethal to cells produced in tissue cultures, and it prevents the growth of several microorganisms across the spectrum (62). *Cylindrospermopsis*, Umezakia, and Aphanizomenon-like cyanobacteria produce these toxins, and those toxins cause kidney and liver failures. Further, it causes tissue failures and destroys the organ (59). Toxins such as Tolytoxin, Tubercidin, Scytophycins, Actiphycins, Indolocarbazoles and tautazoles, microbilinisonitriles, paracyclophanes are belongs to cytotoxins (59, 63).

##### 2.2.1.1 Tolytoxin

Tolytoxin is produced by Tolypothrix, a polyketide macrolide that perturbs the filaments; it can interact with the actin monomers cytoskeletal and inhibit polymerization, enabling intracellular transport and mitosis. Cock and Cheesman (64) in 2023 reported that, with *in vitro* studies, the IC_50_ of tolytoxin is 50–100 nM. The primary mode of action of this toxin is on the apoptotic cell death, preventing polymerization, which is essential in intracellular transport and mitosis in human health. The main mechanism of this toxin is to enhance apoptotic cell death through the disruption of the cytoskeleton. This toxin can be used as an anti-metastatic since it can reduce the cancer cell death (65–67). Tolytoxin has profound effects on the cell shape and microfilament distribution of mammalian cells, and these changes can be produced by very low concentrations of this agent. Tolytoxin inhibits actin such as polymerization *in vitro*, which you would expect to see in a gel, and thereby explains the observed cellular effects (66).

##### 2.2.1.2 Tubercidin

This toxin is an adenosine analog, a purine nucleoside that is incorporated in the synthesis by nucleic acid. The specific toxin of this fungus is capable of RNA translation and transcription, causing eventual apoptosis (68). According to the current literature, it has the potential to be used in clinical trials against fungal invasions such as Candida albicans and Leishmania donovani (68, 69).

##### 2.2.1.3 Scytophycins

Scytonema is a toxin synthesized by cyanobacteria macrolide compounds targeting actin filaments, leading to cell death. This can affect cytotoxic effects against breast cancer cells and leukemia cells (57, 70).

##### 2.2.1.4 Indolocarbazoles

This toxin is an alkaloid group originating from tryptophan (71). It interferes with DNA supercoiling and influences protein kinases, such as topoisomerase I and II (72). Further, it can hinder the transcription by inhibiting the VEGFR and EGFR signaling, which is crucial for angiogenesis and cancer cell division (73).

##### 2.2.1.5 Actiphycins

This toxin is a cyclic peptide containing a significant number of proteinogenic amino acids contributing toward the stability of this molecule. The major use of this is to stop the molecule from replicating through binding to DNA polymerase. Al-Hussieny (59) reported that it is a kind of toxin belonging to cytotoxins. There is only limited documentation regarding these toxins, and further research is needed regarding these toxins.

#### 2.2.2 Dermatoxins

Dermatoxins include aplysia toxins and debromoaplysiatoxin, which predominantly result from contact and are related to cyanobacterial toxins (74). These toxins stimulate the protein kinase C (PKC), which is involved in cell proliferation and differentiation, and promote inflammation in human skin cells. Dermatoxins are poisonous chemicals that cause skin issues, and when the skin is repeatedly exposed to dermatoxins, the PKC remains active for a long time, increasing the occurrence of tumors (75).

#### 2.2.3 Neurotoxins

Neurotoxins are some of the most widely recognized types of microalgae toxins, and they work in the nervous system by interfering with ion channels and neurotransmitters (75). *Alexandrium* species generate Saxitoxin (STX), which binds to voltage-gated sodium channels in both nerve and muscle cells, thus preventing the infiltration of sodium ions into cells; this triggers paralysis and possibly fatal cessation of breathing in humans (76). Likewise, the toxic alkaloid, domoic acid, elaborated by diatoms of the genus *Pseudo-nitzschia*, mimics the neurotransmitter freshwater, seeking out specific glutamate receptors in the central nervous system. This causes excitotoxicity, in which stimulation of glutamate receptors results in neuronal lesions and memory loss, a condition referred to as amnesic shellfish poisoning (77).

##### 2.2.3.1 Neurotoxic alkaloids

Alkaloids are usually lethal and poisonous in a short time as they cause paralysis of respiratory muscles and skeletal muscles, often resulting in respiratory issues and death. *Oscillatoria* and Trichodesmium are producing different forms of these kinds of toxins (78).

Anatoxin: *Anabaena flos-aquae* species produces this toxin, which contains a 765 Da molecular weight (79).

Homoanatoxins: *Oscillatoria rubescens* produces a and is less toxic than anatoxin (79).

Anatoxin-a(s): *Anabaena* produces this toxin, which is ten times more toxic than anatoxin, with a molecular weight of 252 Da (79).

##### 2.2.3.2 Paralytic shellfish poisons (PSPs)

PSPs include 18 toxins that paralyze crustaceans and are classified into three main classes, gongyautoxins, saxitoxin, and C-toxins, usually produced by species such as *Anabaena* circinalis and Aphanizomenon flos-aquae (80). These toxins are thought to have an immediate neurological response due to the interference of nerve impulses by blocking sodium channels, but they do not affect potassium leak currents (81).

#### 2.2.4 Hepatotoxins

Hepatotoxins-producing genera include *Anabaena*, *Microcystis*, *Cylindrospermopsis*, *Nodularia*, *Oscillatoria*, and *Nostoc* (82). Microcystins are the most abundant of the cyanobacterial toxins (75). Nevertheless, they are slower in killing the organisms than neurotoxins, and the process of death can take 5 min to as long as several days, based on the rate of more factors and conditions, such as the type of poison, an animal’s weight, and the dose. These toxins are classified into three groups (82).

The hepatotoxins act on the liver and produce both short-term and chronic effects. Microcystins isolated from M. aeruginosa, for instance, interfere with serine/threonine protein phosphatases PP1 and PP2A; increased intracellular proline-directed serine/threonine phosphorylation induces hepatocyte apoptosis/necrosis (75). This toxin is considered to be very stable; hence, the long-term effects of this algae grown in water bodies are very detrimental to water sources used for human consumption (75, 83). Another similar but distinct toxic compound affecting liver cells is nodularin, which is produced by *Nodularia spumigena* cyanobacteria and is typical for brackish water conditions as well only limited documents are (75).

##### 2.2.4.1 Microcystins

These are monocyclic seven-chain peptides with an unusual resident amino acid (84). Microcystin-LR (MC-LR) inhibits green algae growth by regulating antioxidant and photosynthetic systems by harmful algae (84). This is because the peptide ring includes five amino acids that are involved in the biosynthesis of all the structural variants of microcystins found in this species. Microcystins were found in a stream inhabited by fish of the genus Brook (Salvelinus fontinalis) (85). Some other related species belonging to *Oscillatoria*, *Nodularia*, and *Lyngbya*, as well as genera like *Anabaena*, *Nostoc*, and others, are employed in the production of these materials (84). Among these toxins, only microcystins MC-RR, MC-LR, and MC-YR have been identified. There are often lethal microcystins, such as microcystin molecule weights. The levels of microcystins may be detected as long as 909–1,044 years, depending on the species (85). Microcystins are known for their long-term heat shock and other related features, and it has emerged that they are capable of enduring boiling without denaturation (84). They are stable in terms of pH changes and are freely soluble in water. Ethanol, methanol, and acetone cells will require energy to metabolize the poison (85).

##### 2.2.4.2 Nodularin

MC–LR is slightly similar to this compound; it is a pentacyclic monocyclic peptide, but significantly smaller. The peptide ring has a molecular weight of 824 Da and contains amino acids similar to those found in MC–LR (86). Although a range of varieties have been spotted around the world, only one is manufactured by the *Nodularia spumigena* species, and their growth is toxic to humans and cattle as well as to that of MC-LR (87).

#### 2.2.5 Endotoxins

##### 2.2.5.1 Lipopolysaccharides (LPS)

Lipopolysaccharides are glycolipoproteins present in the cell wall. These chemicals are toxic to humans; when it was injected into the peritoneal membranes at a dose of 1–1.2 mg/kg, they were found to be fatal to rats even at 48 h using *in vivo* experiments (57).

## 3 Overview of microalgae and their uses in food products

Microalgae are successfully included in different sectors such as the food industry, pharmaceuticals industry, biofuel production, wastewater treatments, fertilizers, and cosmetics industry (31, 35, 88, 89). Globally, Microalgae have been recognized as sustainable, healthy, nutritional, and eco-friendly for social development. From ancient times, countries such as China, Japan, and many coastal regions worldwide mainly used microalgae for food production.

*Arthrospira platensis*, *Chlorella vulgaris*, *Dunaliella salina*, *Isochrysis galbana*, *Nostoc sphaeroides*, *Spirulina maxima*, and *Spirulina platensis* are used to produce commercial feed or food products (1, 90, 91). *C. vulgaris* is a famous microalga commercially cultivated to produce beta-carotene, astaxanthin, canthaxanthin, and chlorophyll, which can be used as food ingredients. Microalgae can additionally be used as food additives, bakery products, food supplements, and beverages (92–95). Docosahexaenoic acid is produced by the commercially cultivated *Cryptothecondium cohnii*, *Schizochytrium*, *Thraustochytrium*, and *Ulkenia* (96). Products of microalgae, including cyanobacteria, have been proposed for sterols, proteins, lipids, n-3 and n-6 fatty acids, microalgal oil, hydrocarbons, vitamins, polysaccharides, phycobiliproteins, zeaxanthin, lutein, phycocyanin, phycoerythrin, and antioxidants (96–105).

The protein in autotrophic and heterotrophic cyanobacteria is higher than the protein in pork and beef (104). Further, when we give small amounts of microalgae with animal feed, it improves the nutritional value of the feed and the animal’s performance and enhances the quality of products like meat, milk, and eggs (106). Table 1 clearly shows the microalgae-based food products and their nutritional values. Different value-added products can be commercialized using these microalgae, which is an emerging trend in this field. Asian countries like Sri Lanka still have a hidden fear of using these micro algae and fewer products in the market related to this field. This article suggests the research gaps in the microalgae-related food industry worldwide as one part of the article.

**TABLE 1:** Table of microalgae-based food products and their macromolecules.

Microalgae	Micro algae-based food product	Macromolecules	References
*Haematococcus pluvialis*, *Nannochloropsis gaditana*, *Karlodinium veneficum*, *Isochrysis galbana*, *Chlorella* sp., *Scenedesmus almeriensis*, *Tetraselmis suecica*	Additives	Lipids	(93)
*Arthrospira platensis*, *Chlorella* spp., *Nannochloropsis* spp., *Tetraselmis* sp., *Dunaliella salina*, *Haematococcus pluvialis*, *Porphyridium* sp., *Phaeodactylum tricornutum*, *Scenedesmus* sp.	Biomass	Carbohydrates, protein, lipids	(145, 146)
*Chlorella vulgaris*, *Arthrospira platensis*	3D Printed Cookies Microalgae flour	Proteins	(147–149)
*Schizochytrium* sp.	Fortified beverages	Lipids	(95)
*Arthrospira platensis*, *Chlorella* sp.	Additives	Pigments	(150)
*Scenedesmus almeriensis*, *Isochrysis galbana*, *Nannochloropsis gaditana*, *Tetraselmis suecica*	Wheat bread	Proteins	(151)
*Arthrospira platensis*, *Scenedesmus obliquus*	Chocolate	Proteins lipids carbohydrates	(152)
*Arthrospira platensis*, *Nannochloropsis gaditana*, *Pyrocystis lunula*	Biomass	Carbohydrate	(153)
*Arthrospira platensis*	Yogurt	Proteins	(92)
*Arthrospira platensis*	Chocolate milk	Proteins	(154)
*Arthrospira platensis*, *Chlorella* sp.	Beverages	Proteins, carbohydrate	(155)
*Chlorella* sp., *Arthrospira platensis*	Dietary supplements	Proteins	(94)

## 4 Pathways of toxin contamination in food products in algae

Algae, specifically microalgae, are increasingly in demand in the food market due to their rich nutritional profiles. However, toxin contamination from microalgae is a significant threat to food safety and human health. Algal toxins, which are known as phycotoxins, can accumulate in food chains, impacting human health through various pathways (107). These toxins are critically produced by harmful algal blooms, where specific algae species release toxins as secondary metabolites. Understanding the pathways of contamination is significant for mitigating risks and ensuring food safety regarding microalgae (7). Concerning the pathways of toxin contamination in food products, microalgae are much important, and it is critical to avoid those contaminations in algae as they can cause health risks and environmental pollution.

### 4.1 Direct consumption of toxin-producing algae

Microalgae such as *Microcystis*, *Anabaena*, and *Nodularia* are known to produce toxins like microcystins and nodularins, which can directly contaminate food products when algae are consumed as dietary supplements or functional foods (108). *Spirulina*, commonly used in the food industry, may occasionally be contaminated with toxic cyanobacteria if not adequately monitored when manufacturing dietary products, dessert products, and food additives (109). Cyanotoxins are heat-stable, and conventional food processing methods like pasteurization and cooking are ineffective at eliminating them, thereby posing risks to consumers (110). Research studies regarding this topic is an emerging trend, as there may be some direct consumption of toxin-producing algae which has not yet been identified without knowing.

### 4.2 Bioaccumulation in the food chain

Bioaccumulation is highlighted as another key approach to having algal toxins in seafood and other sea products. Some of these crustaceans and mollusks include mussels and oysters; these are categorized as filter-feeding mollusks that can concentrate toxins that are produced by algae in their tissues (74, 76, 107). Domoic acid, a substance that is transmitted from algae to fish and shellfish, is toxic to mammals, while saxitoxins, which are consumed by fish and bivalves, are also toxic to mammals. Brevetoxins, which move up the food chain from algae to fish, are toxic to humans through the process of biomagnification (74, 107, 111). This bioaccumulation is identified as a major issue for aquaculture, as seafood consumption is rapidly increasing globally. Contaminated water, whether freshwater or seawater, is used in food production, allowing toxins to enter food products. For instance, the use of polluted water for washing food crops, watering crops, or returning yield to feed livestock floods these products with toxic substances (42, 112). Microcystins, prevalent in freshwater systems, can persist in treated water, posing food safety risks when surface water is utilized for irrigation in agricultural regions (113).

### 4.3 Understanding cross-contamination and industrial processing

In the industrial usage of algae products in the food industry, contaminants can potentially spread (74). This can occur when toxic microalgae get mixed with non-toxic microalgae at harvesting time and are processed, or whenever improper washing and storage allow toxins to transfer from one batch to another (76). Contamination can generally be critically high, but an ineffective quality control strategy during algal harvesting or processing can worsen the problem (42). There is a research gap about the effect of the consumption of microalgae directly in food, in contrast to the industrial processing of them.

### 4.4 Global environment and climate change

The harmful algal bloom frequency has increased due to changes in the environment, especially climate change, as a higher risk of contamination with foods (114). Increasing temperature of the water, enrichment by nutrients from the agricultural effluents, and changes in water currents favor the growth of toxic algal strains. As a result, the toxins could suddenly appear in food products that have never been observed in certain areas, thus creating a new challenge for monitoring and controlling the issue (42).

Toxic metabolites and their characterization that cause diseases in microalgae are essential for human health as the incidences of harmful algal blooms are increasing (74). Cyanotoxins, which are representative of toxins created by cyanobacteria, are ingested by people through drinking water and consuming seafood, aerosolized toxins inhaled by people, and skin contact due to water sports, etc (42). Given these pathways of exposure, it becomes pivotal for policymakers to understand the extent of associated health risks to establish a structure for prevention measures (115). It is very important to conduct in-depth studies regarding the effect of global environmental changes and microalgae toxins.

### 4.5 Consumption of water and foods contaminated with toxins

Consumption of water polluted with these toxins is one of the most hazardous ways of getting involved with microalgae toxins (74). Consequently, the toxins of cyanobacteria, primarily microcystins, are the primary worries in freshwater ecosystems where harmful algal blooms threaten water accessibility for drinking. Among the toxins of this genus, microcystins produced by species such as *Microcystis aeruginosa* are the most dangerous because they possess hepatotoxic effects; they inhibit protein phosphatases Protein Phosphatase 1 and Protein Phosphatase 2, cause liver injuries and tumor issues (113, 116). High-level microcystins were shown to cause liver failure upon acute toxicity, while low-dose sub-chronic or chronic toxicity of microcystins affected liver cancer (85). In the study by Hilborn (117). They also note that there is a massive threat of exposure to microcystin in drinking water, as water utilities are still not capable of eradicating the toxins from the drinking water.

In the aquatic environment, another clear danger is the build-up of neurotoxins in seafood. Currently, Paralytic Shellfish Poisoning is caused by toxins named saxitoxins, which are generated by certain species of *dinoflagellates*, including *Alexandrium*, and accumulated by shellfish. Paralytic Shellfish Poisoning can be as mild as tingling and numbness, and severe enough that it leads to paralysis and respiratory failure (74, 118). In the same way, domoic acid, a neurotoxin of diatoms such as *Pseudo-nitzschia*, leads to amnesic shellfish poisoning, which impacts the central nervous system. Amnesic Shellfish Poisoning signs include memory impairment, particularly confusion, short-term memory loss, and, in extreme form, experiential shock, seizures, and death (119).

These poisons in the food chain increase the risk to consumers’ health, particularly for those, including fishermen, who consume marine products. This issue has been well documented, especially in coastal regions, where seafood is a staple in most local diets. This situation has created a need to enhance the surveillance of toxin levels in commercially and recreationally harvested shellfish (74). Inhalation and Dermal Contact Apart from ingestion, the second mode of exposure is inhalation of aerosolized microalgae toxins during games in or near water bodies that contain the toxins (118). Cyanobacterial blooms can produce gas-borne toxins, and these are inhaled, hence causing respiratory manifestations. The aerosolized microcystins can trigger allergies, asthma, and other respiratory problems like headaches, watery or bloody noses, dizziness, vomiting, diarrhea, skin rash, and, in severe cases, hives and pneumonia in children and the elderly (120). Leisure activities such as swimming, boating, and water sports put the individual at a greater risk of inhaling the toxins present in water and or getting in direct contact with the toxins through the skin, which enhances their health perils (118). Microalgae toxins are also dangerous when dermally administered, and this happens especially when one comes into direct contact with the water, such as when swimming (115). The primary effects of the lyngbyatoxins present in the cyanobacteria include skin rash, skin irritation, and skin inflammation (120). The above-presented dermatological symptoms are typical complaints by people who swim in water bodies containing harmful algal blooms (121). While skin contact is usually less dangerous than contacting the substance with the mouth or nose or breathing it in, it still causes severe distress and, with persistent irritation, followed by infection (74). Table 2 depicts the summary of major microalgal toxins in the food products, maximum limits, and their toxicity data.

**TABLE 2:** Summary of main microalgal toxins in food products, cyanobacterial genera, toxicity data, and effect of the toxins.

Toxin type	Specific toxins	Cyano-bacteria genera	Effect of toxins	Contaminated food product	Recommended concentration range	Codex maximum level	Reference dose (RfD)	Lethal dose (LD50)	References
*Cytotoxins*	Cylindro-spermopsin 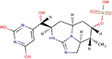	*Cylindro-spermopsis*, *Umezakia*, *Aphanizomenon*	Affects the kidneys and liver, and causes tissue destruction and failure of potential organs	Cyanobacteria and crops	0.1–1.3 μg/L in water; up to 3.8 μg/kg in crops	No Codex limit; WHO provisional: 0.7 μg/L in drinking water [World Health Organization (WHO) (156)]	0.03 μg/kg bw/day (158)	75 to 300 μg/kg (mouse, oral)	World Health Organization (WHO), (157); United States Environmental Protection Agency (US EPA), (158); Al-Hussieny, (59); Grace et al. (25); World Health Organization (WHO) (159); International Agency for Research on Cancer (IARC) (160); World Health Organization (WHO), (161)
*Lipopoly-saccharides (LPS)*	Lipopoly-saccharides (LPS) 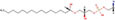	*Cyanobacterial* sp.	Toxic effects for humans cause illnesses and are lethal for mice when injected into the peritoneal membrane	Cyanobacteria	7 to 16 mg LPS per gram of biomass dry weight	No Codex limit	No established RfD	1 to 2 μg (Human)	Al-Hussieny, (59); Grace et al. (25); Skočková et al. (162); Dinges and Schlievert, (163); Stewart et al. (142)
*Hepatotoxins*	Microcystin 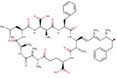	*Oscillatoria*, *Nostoc*, *Microcystis*, *Aphanocapsa*, *Anabaenopsis*, *Anabaena*, *Hapalosiphon*	Directly affect community of zooplankton and affecting species that rely on cyanobacteria as a food source	Cyanobacteria and crops	0.1–1.3 μg/L in water; up to 3.8 μg/kg in crops	No Codex limit; WHO provisional: 0.7 μg/L in drinking water [World Health Organization (WHO) (156)]	0.03 μg/kg bw/day (158)	75 to 300 μg/kg (mouse, oral)	Al-Hussieny, (59); Grace et al. (25); World Health Organization (WHO) (159)
	Nodularin 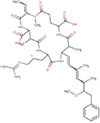	*Nodularia*	Hepatotoxic effects in animals and humans, and Similar effects on zooplankton communities	Water and fish	No universally recommended concentration rate of nodularin in microalgae, but research indicates that it can be present in various concentrations	Not specify a maximum Codex level	WHO drinking water concentration limit for nodularin extended from microcystins-LR) is 1.5 ug/L	Generally reported as 50 to 70 micrograms per kilogram (μg/kg)	Al-Hussieny, (59); Grace et al. (25); Stewart et al. (164); Chen et al. (165); Bownik (166); World Health Organization (WHO) (143)
*Neurotoxins*	Saxitoxin 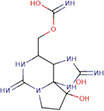	*Lyngbya*, *Anabaena*, *Aphanizomenon*	Causes paralytic shellfish poisoning (PSP), cause respiratory failure and death	Shellfish (mussels, clams, oysters), crustaceans	1.5–2 μg STX equivalents/kg	Codex: 800 μg STX-eq/kg in shellfish meat (167)	0.0007 μg/kg bw/day (US EPA)	10 μg/kg (mouse)	Al-Hussieny, (59); Grace et al. (25); European Food Safety Authority (EFSA) (168); United States Environmental Protection Agency (US EPA) (158)
	Anatoxin-a(s) 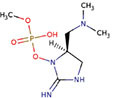	*Aphanizomenon*, *Cylindrospermopsis*, *Lyngbya*, *Anabaena*	Affects neurotransmitter activities and leads to death by respiratory issues.	Cyanobacteria like *Spirulina*	The Office of Environmental Health Hazard Assessment (OEHHA) recommends a short-term notification level of 4 micrograms per liter (μg/L) of drinking water.	No Codex limit; The Codex Alimentarius Commission has not established a specific Codex maximum level (ML)	Reference dose (RfD is 3 μg kg per day	250 μg/kg body weight	Al-Hussieny, (59); Grace et al. (25); Chorus and Bartram (169); World Health Organization (WHO) (157)
	Homoanatoxin-a 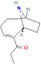	*Oscillatoria*, *Anabena*	Respiratory muscles were paralyzed to respiratory muscles and similar to Anatoxin-a.	Cyanobacteria	No universally recommended concentration maximum drinking water standard of 6 μg/L-according to some guidelines provisional standard of 2 μg/L-New Zealand WHO-30 μg/L for short-term exposure 60 μg/L for recreational water exposure	No Codex limit no specific Codex maximum level (ML)	No established RfD	112–225 mg and 1,125–2,250 mg of freeze-dried algal material per kg human body weight	Al-Hussieny, (59); Grace et al. (25); Bruno et al. (170); Lilleheil et al. (171); Zhang et al. (172)
	Brevetoxins 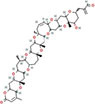	*dinoflagellate Karenia brevis*	Wheezing, asthma, and respiratory distress	Shellfish, especially in Florida and Mexico	20–300 μg/kg in shellfish	Codex: 200 μg/kg	0.002 μg/kg bw/day (US EPA)	455 μg/kg (mouse)	Food and Drug Administration (FDA) (173); Poli et al. (174); Amzil et al. (175); Fleming et al. (176)
	Domoic acid 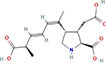	*Pseudo-nitzschia*	Vertebrate central nervous system and other glutamate receptor-rich organs, memory loss gastrointestinal distress, confusion, disorientation, coma, and death	Shellfish (mussels, clams), finfish, crabs	20 mg/kg in shellfish	Codex: 20 mg/kg (shellfish meat (EU)	0.075 mg/kg bw/day (US EPA)	35 and 70 mg/kg (mouse, oral)	European Food Safety Authority (EFSA) (177); Bates et al. (178); Lefebvre and Robertson, (179)
	Anatoxin-a 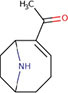	*Cylindrospermum*, *Anabaena*, *Phormidium*, *Aphanizomenon*, *Oscillatoria*, *Microcystis*	Affects the respiratory muscles, and as a result difficult to breathe and death.	Freshwater fish, shellfish, and possibly drinking water	Rarely quantified in food; mostly in water at 0.1–60 μg/L	No Codex limit	No established RfD	200–375 μg/kg (mouse)	Al-Hussieny, (59); Grace et al. (25); Aráoz et al. (180); Chorus and Bartram (169); World Health Organization (WHO) (157); Van der Merwe, (181); Polhemus and Zeise, (182); Bruno et al. (170)

## 5 Impact of microalgae toxins on human health

A current concern arising from microalgal toxins is hepatotoxicity and gastrointestinal illness, often linked to microcystins synthesized by the cyanobacterium *Microcystis aeruginosa* (122). A detailed illustration showing the impacts is shown in Table 2. Microcystins are very resistant and tend to concentrate in water reservoirs, and their ingestion through contaminated food or drinking water due to algal blooms poses a threat to the liver (85, 123). Symptoms of toxicity/exposure, acute dermal and oral exposure result in vomiting, abdominal pain, and nausea, while over-exposure to chronic systemic conditions results in hepatotoxicity, tumor formation, among other diseases due to its strong hepatotoxicity (124).

This toxin is also very stable, and normal water treatment systems may not fully eliminate the microcystins, presenting a continued threat to communities using such water (122). In addition to being toxic, these microalgae exhibit neurotoxic influences—saxitoxins and domoic acid- causing manifold health risks (85). The toxins that cause paralytic shellfish poisoning (PSP) include saxitoxins, which are produced by *Alexandrium* and other species and result in a tingling sensation to paralysis and failure in respiration (125). Another neurotoxin that is closely associated with species including *Pseudo-nitzschia* is domoic acid, which can cause amnesic shellfish poisoning (ASP), which affects the hippocampus in the brain, impairing memory and learning capability (126). These neurotoxicity effects can be much worse in some susceptible categories of the population, such as children, the elderly, and immunocompromised persons (125).

Transmission pathways and second-order threats, besides other types of exposure (for instance, bioaccumulation in fish products), are also considered to threaten public health (122). Hazardous toxins can be found in fish, shellfish, and other seafood; those toxins bioaccumulate and move up the food chain toward human consumers (127). This bioaccumulation is somewhat different from other toxin concentrations as toxins may remain in the seafood even when there are no signs of algae blooms evident (128). For instance, surveillance schemes in the Baltic Sea have established that microcystin toxins may become incorporated within fish tissues, thereby posing risks through the consumption of contaminated fish outside the bloom season (124).

Thus, where waterborne exposure to microalgal toxins may be possible, toxic compounds are also aerosolized in coastal environments, posing significant risks to respiratory health (122). Research has shown aerosolized cyanotoxins, which, once inhaled, can penetrate the respiratory system, resulting in respiratory inflammation, asthma, and chronic lung disease (129). This form of exposure mainly affects coastal people and those who work closely with water, such as lifeguards and fishers (127).

According to the available documentation, there are clinical records regarding the food poisoning events caused by the contamination of microalgal toxins in different countries. As shown in Hinder et al. (55), 56 patients were identified with various toxic effects while 6 patients died due to the consumption of seafoods containing noxious substances, with ages 5–94 years, from 1998 through 2009 in the United Kingdom (55). Kim et al. (130) also show that with the available data of the Asia-Pacific Countries, shellfish that accumulate toxins when ingested by humans can cause diverse symptoms (diarrhea and muscle pain) and even death (130). Based on the available literature data till 2024, it clearly shows that six allergic reactions, at least 70 illnesses, and 14 mortality records have been globally recorded (131). As mitigation strategies are better than the cure, studies regarding these microalgal toxins is a timely action.

Public Health and Management Implications showed the different consequences of microalgal toxins and also pointed to a dire need to come up with ways to tackle the issue, given that it has both long- and short-term effects on the health of those who consume them. Toxic microalgal monitoring surveillance programs for water, seafood, and coastal atmosphere are crucial in preventing microbial access to communities (74). Innovations like hand-held optical equipment relevant to the remote detection of toxin-producing algae at the bloom formation scene could decrease exposure dangers (123). Furthermore, raising awareness of how to avoid consuming seafood contaminated by hazardous algal blooms or toxins can considerably decrease people’s exposure (132). Consequently, microalgal toxins are widely adverse and have numerous effects that relate to human health, and they can affect all age groups regardless of direct or indirect contact. Since climate change poses a potential threat to hazardous algal bloom, observing and explaining activities coupled with technological advancement can help to manage health-related hazards, as shown in Table 2 (85). More efforts are required in the cross-disciplinary analysis of toxicity to garner more information on the toxicity, diagnostic techniques, and prevention measures that would protect particular risk groups (85).

## 6 Detection and mitigation strategies

Humans are currently at a significant risk from the HAB, due to rising seafood consumption. Consuming contaminated fish, seafood products, or water could expose humans to harmful toxins produced by HABs causing respiratory illness, memory loss, seizures, digestive tract problems, and skin irritation and also fatalities (133). Due to this possible impact of HAB, the detection of harmful microalgal toxins has become essential for human health protection because which provides a major risk to human health (134).

There are different mitigation strategies to eliminate the extracellular toxins present in the microalgae. Different technologies utilize chemical and physical methods, such as activated carbon adsorption and membrane filtration, as well as chemical inactivation through the application of oxidants like chlorine, potassium permanganate, ozone, or ultraviolet light (135). These approaches harness the inherent abilities of diverse microorganisms, such as macrophytes, microalgae, macroalgae, bacteria, viruses, actinomycetes, and pathogens, to regulate HABs (136). Effective monitoring and early identification are essential for controlling risks from aquatic environments, toxin-producing microalgae. Moreover, regulation of nutrients, specifically carbon, nitrogen, and phosphorus, is considered an important long-term strategy for preventing the formation of hazardous blooms, which are made worse by eutrophication (137).

### 6.1 Detection methods

In recent times, there are different types of detection methods such as chemical methods, biochemical methods, molecular methods, biosensors to detect the microalgal toxins. LC-MS/MS, HPLC, HPCE are the chemical techniques utilized for the detect aquatic algal toxins. These methods have separation efficiency, low solvent cost, small sample volume, and ability to detect multiple toxin groups. However, these have limitations such as high technical complexity, high cost, long operating time, and requires an expert in the field to operate. Biochemical assays such as PPIA, ELISA, and cell-based assays provide specificity, sensitivity, and speed (134). Compared to traditional microscopic identification and numeration methods, molecular method like quantitative PCR (qPCR) allows for the simultaneous amplification and detection of specific DNA sequences, and its objectivity, sensitivity, and specificity make it suitable for routine monitoring of toxic algae (138). as well as high sensitivity, quick turnaround time, resilience, affordability, ease of use, accuracy, and low power needs of biosensors offer attractive options to get beyond the limitations of traditional detection quantification techniques (133).

### 6.2 Public awareness

Public awareness campaigns can significantly minimize human exposure to cyanotoxins either due to recreational activities, drinking untreated water or consuming seafood. In addition, limiting the risk of bacterial bloom formation can be achieved by implementing good social practices and avoiding the disposal of organic and inorganic waste near water sources. Under nationally supported programs in developing countries, media coverage of how climate change affects the safety of food ingested could be implemented. It is an urgent need to strengthen the aware the children and the public community about these microalgal toxins from their early childhood, because for children, it can lead to an intellectual disability due to their poisonous effect. In addition to knowledge sharing sessions, such as workshops, global networks programs, and research discussions to aware the community can be identified as a timely need to mitigate this microalgae toxin. Moreover, governmental and non-governmental organizations should interfere in the microalgae-related toxins, and they can organize awareness programs (139). In contrast, regulations should be established by governments to make sure that undesirable industrial effluents are correctly cleaned up before they enter aquatic bodies. Only awareness is not applied, or restrictions on recreational activities, or the prohibition of any water-related activity, depend on the detected levels of the monitored toxin. Indeed, having a categorical risk to cyanotoxins classification (low, medium, and high) will help choose the appropriate action (135).

### 6.3 Consumer protection and regulatory models

In 1998, the WHO established a provisional TDI for chronic exposure to MC-LR of 0.04 g/kg body weight and a provisional guideline value of 1 g/L in drinking water (cell-bound and extra-cellular toxins) to protect the public from the harmful effects of cyanotoxins. For MC-LR, CYN, STXs, and ANTX, WHO suggested revised provisional guidelines in 2020. To better reflect the health effects, temporary guidelines were modified. However, the provisional TDI remained the same for MC-LR.

It has been suggested that to measure the risk accurately, the concentration of MCs present should be considered. A new provisional recommendation of 12 g/L has been recommended for short-term exposure, but the 1 g/L value for drinking water is used for long-term exposure. Due to a lack of long-term toxicological evidence, ANTX was only given a provisional guideline value of 30 g/L for short-term exposure in drinking water. In contrast, three provisional guideline values have been assigned to CYN. Guidelines for drinking water exposure have been established at 0.7 g/L for short-term exposure and 3 g/L for long-term exposure. Provisional standards for drinking water at 3 g/L have been sent to STX (135).

Certain groups of people are more affected than others regarding the adverse health effects of microalgae toxins, such as children, the elderly, persons with chronic diseases, or other compromised health conditions of the body (74). Therefore, there is potential for high exposure to people living in areas where the dependent water resources are surface waters or in areas where seafood is a staple food (7). Current findings indicate that Dichlorobenzene exposure at sub-chronic levels and below the toxicity level may also lead to chronic health issues. Including liver cancer and neurological disorders (116). An area of special interest is the ability of microalgae toxins to act as environmental carcinogens. Researchers have identified that deficient proteins in cells regulated by microcystins enhance the growth of liver tumors (85). Acute exposure to low-concentration toxins can integrate over time and cause cumulative injuries. Hence, populations with long-term exposure are at a higher propensity to have cancer-related ailments (112). When considering all these mitigation strategies, it shows that public/consumer awareness and regulatory models are the best strategies to mitigate this microalgal toxin for humans.

## 7 Future direction

Despite extensive research, several knowledge gaps hinder our comprehensive understanding of microalgae toxins in food products and their implications for human health. These gaps limit the ability to develop effective mitigation strategies and ensure food safety. Considering these gaps, conducting more studies in the future is essential.

Regarding the production of toxins, several microalgae species are still not fully understood and it is a timely need to research in this field. Toxins such as domoic acid, saxitoxins, and microcystins are commonly identified (140) but there may be others that are unidentified, especially in lower-studied microalgae species. So, it is important to thoroughly profile different microalgae species for potential toxins, which is a highlighted research gap. Also, the production of toxins is significantly influenced by environmental factors, including temperature, salinity, light intensity, and nutrient availability. Prediction and monitoring endeavors are made more difficult by the incomplete understanding of the specific conditions and mechanisms causing this variability. So, clarifying the environmental factors that contribute to the production of toxins requires multidisciplinary research that integrates oceanography, climate science, and microbiology. Also, to monitor changes in the patterns of algal blooms and their toxin profiles, long-term monitoring programs should be established in the location.

There are significant gaps in the knowledge of these toxins’ bioconcentration, bioaccumulation, and bioamplification, as well as the impact of detoxication and covalent binding of microcystins on transfer in the food web, despite the abundance of fundamental data regarding their concentrations in freshwater food webs.

Different detection techniques have been developed to detect microalgae toxins to date, but currently detection techniques frequently lack the sensitivity, specificity, and efficiency necessary for regular monitoring of various toxin types. In general, different detection techniques are appropriate for other purposes (141). So, toxin detection can be improved by advances in biotechnology and analytical chemistry. Furthermore, methods like biosensors, high-resolution mass spectrometry, and assays based on nanoparticles should be improved for rapid, accurate, and cost-effective toxin screening, which are emerging requirements that show the knowledge gaps in this field.

## Conclusion

Even though there is a huge demand for microalgae-incorporated food and dietary supplements in the community, there is still a risk of microalgal toxin poisoning via these food products. As these toxins can cause life-threatening health issues in both humans and animals, it is important to identify these toxin-producing microalgal species, the types of toxins they produce, their biochemical pathways, and the environmental and population factors that influence toxin production. Moreover, understanding the exposure pathways to these toxins and their mode of action is crucial to avoid and treat associated health implications. Apart from that, these microalgal toxins cause critical damage to the environment and the economy. Hence, developing effective detection and mitigation strategies is essential to fight against microalgal toxins. Effective monitoring and early detection of microalgal toxins in natural ecosystems can drastically reduce the risk of human exposure to microalgal toxins. Raising public awareness is also important to address the root causes of HABs. Additionally, imposing regulations as those established by the WHO plays a major role in setting guidelines for safe exposure levels in food and water. The above study shows the need to conduct multidisciplinary research strategies to prevent microalgal toxin contamination and mitigation techniques under commercial food production regarding microalgae.
